# A Robust Method to Quantify Cell Wall Bound Phenolics in Plant Suspension Culture Cells Using Pyrolysis-Gas Chromatography/Mass Spectrometry

**DOI:** 10.3389/fpls.2020.574016

**Published:** 2020-09-09

**Authors:** Lindsey M. Kline, Priya Voothuluru, Scott C. Lenaghan, Jason N. Burris, Mikhael Soliman, Laurene Tetard, C. Neal Stewart, Timothy G. Rials, Nicole Labbé

**Affiliations:** ^1^Center for Renewable Carbon, University of Tennessee, Knoxville, TN, United States; ^2^Department of Food Science, University of Tennessee, Knoxville, TN, United States; ^3^Center for Agricultural Synthetic Biology, University of Tennessee Institute of Agriculture, Knoxville, TN, United States; ^4^Department of Plant Sciences, University of Tennessee, Knoxville, TN, United States; ^5^Nanoscience Technology Center, Department of Physics, University of Central Florida, Orlando, FL, United States

**Keywords:** pyrolysis, gas chromatography/mass spectrometry, lignin, switchgrass, cell suspension cultures, phenolics, chemical composition, lignification

## Abstract

The wide-scale production of renewable fuels from lignocellulosic feedstocks continues to be hampered by the natural recalcitrance of biomass. Therefore, there is a need to develop robust and reliable methods to characterize and quantify components that contribute to this recalcitrance. In this study, we utilized a method that incorporates pyrolysis with successive gas chromatography and mass spectrometry (Py-GC/MS) to assess lignification in cell suspension cultures. This method was compared with other standard techniques such as acid-catalyzed hydrolysis, acetyl bromide lignin determination, and nitrobenzene oxidation for quantification of cell wall bound phenolic compounds. We found that Py-GC/MS can be conducted with about 250 µg of tissue sample and provides biologically relevant data, which constitutes a substantial advantage when compared to the 50–300 mg of tissue needed for the other methods. We show that when combined with multivariate statistical analyses, Py-GC/MS can distinguish cell wall components of switchgrass (*Panicum virgatum*) suspension cultures before and after inducing lignification. The deposition of lignin precursors on uninduced cell walls included predominantly guaiacyl-based units, 71% ferulic acid, and 5.3% p-coumaric acid. Formation of the primary and partial secondary cell wall was supported by the respective ~15× and ~1.7× increases in syringyl-based and guaiacyl-based precursors, respectively, in the induced cells. Ferulic acid was decreased by half after induction. These results provide the proof-of-concept for quick and reliable cell wall compositional analyses using Py-GC/MS and could be targeted for either translational genomics or for fundamental studies focused on understanding the molecular and physiological mechanisms regulating plant cell wall production and biomass recalcitrance.

## Introduction

Rapidly growing efforts to achieve energy sustainability have led to considering lignocellulosic feedstocks as a promising source for the production of renewable fuels and value-added chemicals and products. However, the path to wide-scale production of those from lignocellulosic feedstocks, in general, has been hampered by the lack of cost-effective methods to overcome the natural recalcitrance of biomass prior to conversion (Vanholme et al., 2010; Wright and Turhollow, 2010). This bottleneck is due, in part, to the paucity of tools available to understand the underlying molecular and physiological mechanisms within cell walls that contribute to biomass recalcitrance. While multiple factors contribute to recalcitrance, lignin is widely accepted as one of the main components that prevent the effective enzymatic digestion of cellulose and hemicellulose. Lignin is a complex phenolic polymer composed of syringyl (S), guaiacyl (G), and *p-*hydroxyphenyl (H) monolignol units and is the second most abundant source of renewable and sustainable carbon behind cellulose (Evans and Milne, 1987; Izumi et al., 1995; Lopes et al., 2011). Furthermore, research has shown that lignin S/G ratio significantly affects the recalcitrance of biomass (Chen and Dixon, 2007; Lynd et al., 2008; Tschaplinski et al., 2012). As such, considerable interest has emerged in developing genetically modified biomass such as hybrid poplar and switchgrass with altered cell wall chemistry. These genetic modifications have been achieved through silencing genes involved in the lignin synthetic pathway or overexpressing the transcription factors that repress the pathway. Previous studies in switchgrass have demonstrated that the silencing of caffeic acid 3-O-methyltransferase (*COMT*), 4-coumarate coenzyme A ligase (*4CL*), and cinnamyl alcohol dehydrogenase (*CAD*) led to a decrease in the total lignin content, alteration in the S/G ratio, and improved biomass conversion (Fu et al., 2011a; Fu et al., 2011b; Xu et al., 2011). While the effect of down-regulating *CAD* and *4CL* on biomass yield has not been characterized (Fu et al., 2011b; Xu et al., 2011), silencing of *COMT* was reported to have no effect on biomass yield in both greenhouse (Fu et al., 2011a) and field trials (Baxter et al., 2014). However, down-regulating COMT has been associated with an increase in fermentation inhibitors and phenolic compounds that inhibit simultaneous saccharification and fermentation by *Saccharomyces cerevisiae* (Klinke et al., 2004; Tschaplinski et al., 2012). The overexpression of the R2-R3 MYB4 transcription factor has also demonstrated a significant reduction in lignin and increase in saccharification efficiency, without the need for acid pretreatment (Shen et al., 2012; Shen et al., 2013a; Shen et al., 2013b). While this modification has shown promise in biomass conversion, only one of eight lines survived the first winter in field trials (Baxter et al., 2015). Based on these studies, the potential for genetic modifications to enhance biomass conversion in switchgrass has been demonstrated, but progress in the development of chemically-modified lignin transgenic plants lingers.

A major obstacle for the rapid selection of transgenic plants for reduced recalcitrance is the need to fully regenerate plants in order to screen for altered cell wall chemistry as conventional analyses require mature plants as well as a significant amount (>50 mg) of tissue for each measurement. While transformation and antibiotic selection are conducted at the cell or callus stage, screening for cell wall chemistry is usually conducted when the plant has matured in the greenhouse, leading to a significant delay (≥6 months) between transformation and subsequent analysis. After reaching maturity, the amount of sample necessary for standard wet chemistry methods using sulfuric acid (NREL, 2010), acetyl bromide (Hatfield and Fukushima, 2005), and nitrobenzene (Lopes et al., 2011) is in the 50- to 300-mg range. While these sample sizes can be readily achieved in a biomass setting, it is not feasible to generate such large sample sizes with a cell suspension system.

For these reasons, the goal of this work is to develop a rapid assay to characterize developing cells during the initiation of lignification, in addition to quantify the lignin-precursors content and associated S/G ratio. Previous works have studied early plant cell suspensions and callus cultures to monitor the secondary cell wall formation, cell wall, and extracellular lignin formation (Blee et al., 2001; Kärkönen et al., 2002; Uzal et al., 2009; Kärkönen and Koutaniemi, 2010). Additionally, other studies have demonstrated the feasibility of lignin characterization in switchgrass suspension cultures after induction for initiation of lignification (Shen et al., 2013b), providing support for this strategy. However, these studies used the standard methods for lignin quantification that need significant quantity of samples.

Pyrolysis followed by gas chromatography and mass spectrometry (Py-GC/MS) analysis is a thermochemical technique that has been utilized to study various plant tissue materials. It was used to investigate the structure of lignins (van der Hage et al., 1993; Izumi and Kuroda, 1997), quantify monomeric units of phenylpropanoid-, hydroxycinnamic acid-, and carbohydrate-containing macromolecules (Evans and Milne, 1987), compare lignocellulosic biomass (Izumi et al., 1995; Rencoret et al., 2011; Ross and Mazza, 2011), and capture genotypic difference in lignin composition (Lopes et al., 2011; Gerber et al., 2016), to cite a few examples. Here, to address the limited sample sizes, Py-GC/MS analysis was utilized for the determination of lignin-precursors content in the cell samples prior to and after the addition of epibrassinolide to induce lignification. In addition, all samples were analyzed using standard lignin quantification assays (acetyl bromide and acid hydrolysis) and the syringyl/guaiacyl lignin ratio was determined using nitrobenzene assay as described in previous protocols (Hatfield and Fukushima, 2005; NREL, 2010; Fukushima and Kerley, 2011; Lopes et al., 2011). The results reveal that Py-GC/MS analysis, conducted with about 250 µg of tissue sample as opposed to the 50–300 mg of tissue needed for other methods, provided biologically relevant data, which are comparable to acid-hydrolysis, i.e., the most acceptable method for cell wall compositional analysis (NREL, 2010). Our approach affords a proof-of-concept for rapid and reliable cell wall compositional analyses using Py-GC/MS. We expect that the method could benefit translational genomics and fundamental studies focused on understanding the molecular and physiological mechanisms contributing to biomass recalcitrance.

## Materials and Methods

### Establishment and Induction of Switchgrass Cell Suspension Cultures and Protoplasts

Switchgrass suspension cultures were induced from embryogenic callus developed from inflorescences, as previously described (Burris et al., 2009). Briefly, tillers from the ST1 line of switchgrass were grown in the greenhouse to the E2 to E4 stage prior to collection of inflorescences. After collection, the inflorescences were sterilized for 35 min in 75% commercial bleach supplemented with 1% Tween 20. Inflorescences were then cut longitudinally and placed on Murshige and Skoog (MS) media with benzyladenine (BA) and incubated at 25°C in the dark for 10 days. After incubation in MS, the callus was transferred to LP9 media, specialized media used for switchgrass tissue culture (Burris et al., 2009) for further culturing. Establishment of the switchgrass suspension cultures was carried out following the methods of (Dutta Gupta and Conger, 1999) using the ST1 callus as the initial inoculum. Suspensions were maintained by transferring 5 ml of culture to 15 ml of fresh media at 2-week intervals and maintained in liquid culture medium [MS medium + 9 μm 2,4-dichlorophenoxyacetic acid (2,4D) + 4.4 μm 6-benzylaminopurine (6-BA) + 3% maltose]. Cultures were washed and resuspended in induction medium [0.2 μM epibrassinolide (BL) + 0.9 μM 2,4-D + 4.4 μM 6-BA + 3% maltose) to induce lignification. Aliquots of the cultures were harvested 7 days after transfer to induction or control media. ST1 protoplasts were produced from suspension cultures, as described previously (Burris et al., 2016).

### Extraction of Cells Prior to Cell Wall Characterization

Sample pretreatment and extraction of media and other non-structural cell components were completed to reduce background interference from the carbohydrates-rich growth media and removal of nonstructural phenolics. The cells were vacuum filtered with double Whatman No. 1 filter papers while lightly washing with deionized water (≤10 ml/75 g fresh weight), then extracted with 80% methanol (10 ml/2.5 g fresh weight) in 50-ml centrifuge tubes for 1 h at 80°C. The cells were centrifuged at 10,000 rpm at 25°C, and supernatant collected and combined for two extractions. The extracted pellets were air dried overnight and ground to a powder with a mortar and pestle and stored at room temperature. Samples to be analyzed for chemical composition were subsequently extracted with a Dionex (Sunnyvale, CA) accelerated solvent extractor (ASE) 350. In this process, 15 g of methanol-extracted and air dried cells containing ≤10% moisture were added to a 33-ml extraction cell and sequentially extracted with pressurized water then a mixture of water:toluene:ethanol (2:1:1, *v/v*) under 1,500 psi, 100°C, 5-min heating time, 7-min static time, with three static cycles per solvent mixture. The water used in all analytical work was purified by a Millipore (Billerica, MA, USA) Synergy Ultrapure water purification system. The cells were dried at 40°C overnight and ground with a mortar and pestle.

### Raman Imaging of Switchgrass Suspension Cell Walls

Raman confocal platform (Witec alpha300 RA; excitation wavelength, 532 nm) was used to perform imaging and spectroscopy measurements on the cells, under ambient conditions. The data presented were obtained with a Zeiss 20× objective (illumination/collection). Two spectra (or “pixels”) per micrometer were acquired for the Raman maps. All spectra were collected with a 600 g/mm grating and integration time of 1 s. The Raman maps were constructed by measuring the intensity of the peak at 1,588 cm^−1^ at each point using the data analysis toolbox available in the Witec Suite software package under Project Four. Further data analysis was performed such as partial data averaging and cluster analysis to study local variations in the cell wall content.

### Acetyl Bromide Quantification

The acetyl bromide lignin concentration in the cells was determined following published methods (Fukushima and Kerley, 2011). In brief, 10 ml of 25% acetyl bromide/acetic acid were added to 100 mg of lyophilized extracted cells and the solution was digested at 50°C for 2 h with occasional mixing. After cooling to room temperature, samples were centrifuged at 3000 × g for 15 min, and 0.5 ml was transferred to a tube containing 6.5 ml of acetic acid and 2 ml of 0.3 M NaOH. The solution was then vortexed and 1 ml of 0.5 M hydrochloride was added to the mixture. All measurements were made in triplicate, including a blank and National Institute of Standards and Technology (NIST) Wheat Straw. Absorption scans were collected with a Thermo Scientific (Waltham, MA) Genesys 10S spectrophotometer between 190 and 400 nm with 1-nm spectral resolution, using the absorbance at 280 nm with an extinction coefficient of 23.077 (g/L) for quantification of lignin components.

### Wet Chemistry Chemical Compositional Analysis

The determination of the chemical composition of the cells was achieved by following the protocols developed by the US-DOE-NREL with three replicates (NREL, 2010). A two-stage acid hydrolysis with sulfuric acid (72%, Sigma-Aldrich) was used to fractionate 300 mg of the extracted cells into soluble and insoluble matter. The two fractions were separated through vacuum filtration and ceramic fine porosity filtering crucibles, followed by gravimetric and instrumental analyses. The insoluble fraction consisted of acid-insoluble lignin and ash. Acid-insoluble lignin was determined gravimetrically after combustion of the residue at 575°C for 24 h. The acid-soluble lignin content was measured using a Thermo Scientific Genesys 10S spectrophotometer at 205 nm, and this value combined with the gravimetric value for acid-insoluble lignin accounted for total lignin content. Carbohydrates were quantified using a Flexar high-pressure liquid chromatography system (HPLC) (Perkin Elmer, Shelton, CT) with a Bio-Rad HPX-87P carbohydrate column with a guard column at 85°C using water as the eluent at 0.25 ml/min, with refractive index (RI) detection at 50°C, and a 20-μl injection volume.

### Lignin Composition by Nitrobenzene Oxidation

Following methods by (Lopes et al., 2011), 200 mg of extracted cells were transferred to 5 ml glass tubes with 2 ml 2N NaOH and 200 µl of nitrobenzene, sealed with PTFE caps and heated to 170°C for 3 h. After cooling, excess nitrobenzene was extracted three times with 1-ml ether and the residue acidified with concentrated HCl to pH 2.0. Phenolic compounds were extracted with diethyl ether, dried under nitrogen stream, and re-dissolved in 50% methanol. Separation of phenolics was achieved *via* a Perkin Elmer Flexar HPLC with a photo diode array detector (PDA). The system was equipped with a Prevail C_18_ column (250 mm × 4.6 mm, 5µm particle size, Alltech Assoc., Deerfield, IL) and 10-µl injection volume and eluent of 95% water (pH 2.0 adjusted with acetic acid) and acetonitrile 5% v/v at 1 ml/min. A gradient run was created where acetonitrile was increased from 5% to 15% v/v over 10 min, 20% over 30 min, and to 60% over 40 min. Standards used for identification and quantification included *p-*hydroxybenzoic acid, vanillic acid, syringic acid, ferulic acid, coumaric acid, *p-*hydroxybenzaldehyde, vanillin, and syringaldehyde, all purchased from Sigma (Sigma-Aldrich Corp., St. Louis, MO).

### Py-GC/MS Classification and Quantification

The quantification of lignin-precursors in the cells was performed using the Frontier (Fukushima, Japan) EGA/Py-3030D pyrolyzer. Approximately, 250 µg of the extractives-free cells were weighed into stainless steel cups, pyrolyzed for 12 s at temperature varying from 375 to 500°C to initiate fragmentation of the biomass components and analyzed with a Perkin Elmer Clarus 680 Gas Chromatograph coupled with an Elite 1701 MS (cross bond 14% cyanopropylphenyl, 86% methylpolysiloxane) capillary column (60 m × 0.25 mm ID ×0.25 µm film thickness) with a 80:1 split ratio. The column oven temperature was held at 50°C for 4 min and ramped at 5°C/min to 280°C, held for 5 min, for a total run time of 55 min. A Clarus SQ 8C Mass spectrometer was used in conjunction with a NIST library for identification of the evolved components. Peaks were classified using the mass:charge ratio (*m/z*) in conjunction with the NIST library to distinguish between peaks originating from structural components in the cell (i.e., carbohydrates or lignin) or any residual non-structural compounds from the cells or derived from the cell media. To quantify lignin, syringyl (S), guaiacyl (G), and *p*-hydroxyphenol (H) lignin percentages, the pyrograms were integrated with the TurboMass software v6.0 (Perkin Elmer) and only peaks with significant areas (relative to a 5:1 signal to noise ratio) were summed. Peaks assigned as non-structural or otherwise unidentifiable were removed from the analysis. Peaks categorized as lignin fragments were summed, calculated as a percent of the area of total structural peaks for total lignin-precursors content, and as a percent of lignin peaks for % S, % G, and % H for characterization of the lignin structure. The NIST library identification and peak assignments were cross-validated by referring to primary literature (Evans and Milne, 1987; Izumi et al., 1995; Lopes et al., 2011). All measurements were completed with a minimum of five replicates, including a blank and NIST Wheat Straw (Reference material 8494). Similar to other chromatographic methods, some slight shifts in retention times are observed between replicates. To remove this variation from the statistical analysis, we selected all the peaks (~200) that had a minimum S:N ratio of 5:1, assigned a peak number corresponding to their order based on retention time. Between replicates, we verified that the m/z value for a given peak was the same before assigning the same number to a peak. Principal component analysis (PCA) was performed on the Py-GC/MS peak integration values using the Unscrambler v.9.0 statistical software (CAMO Software, Inc., Woodbridge, NJ) following methods described in previous works (Martens and Naes, 1989; Labbé et al., 2006).

## Results and Discussion

### Chemical Characterization of Switchgrass Cell Suspensions Before and After Induction for Lignification

**Figure 1:**
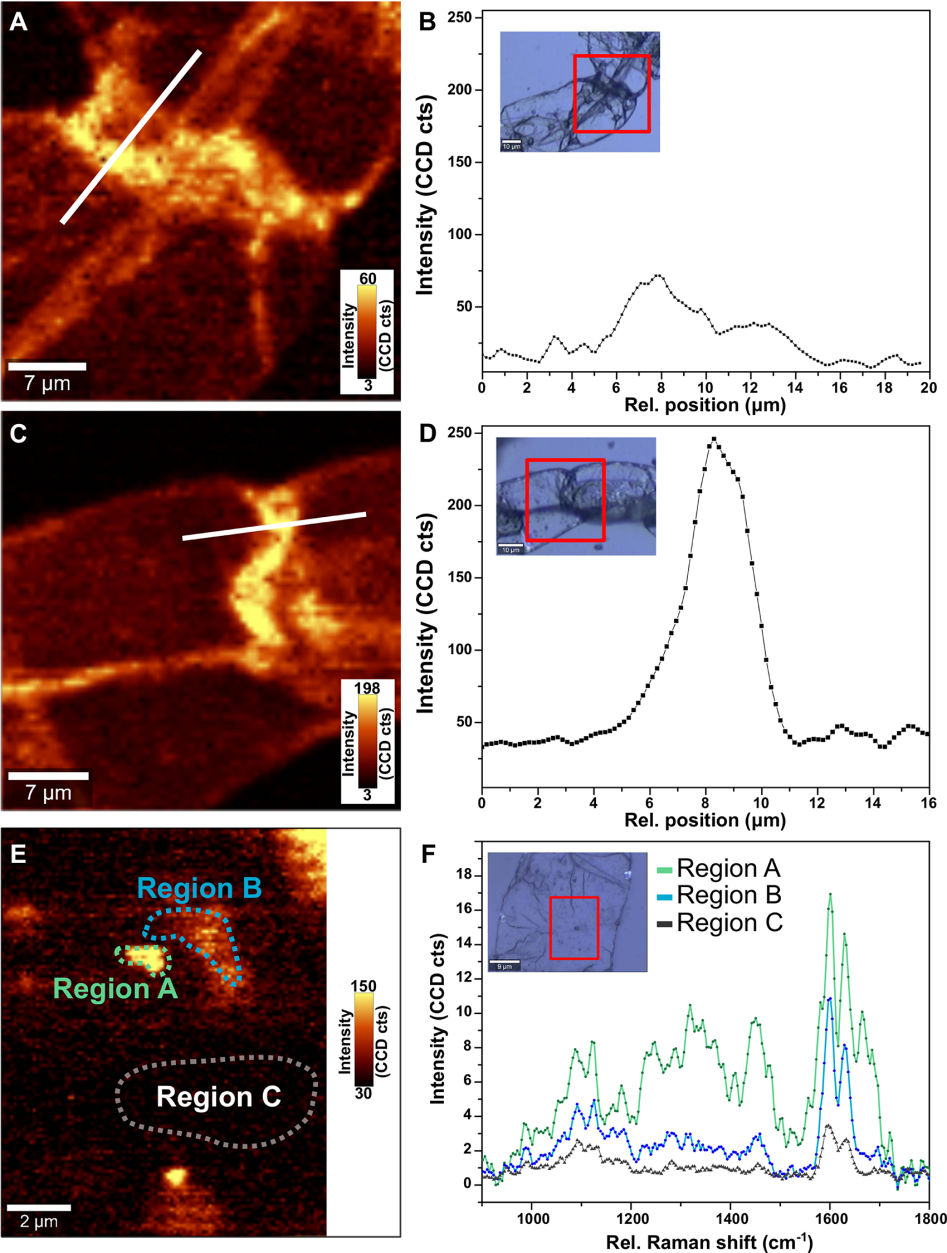
Raman imaging and spectroscopy of the switchgrass suspension cell cultures of uninduced cells **(A, B, E, F)** and **(C, D)** induced cells. Raman maps of the intensity of the peak at 1,580 cm^−1^ for uninduced cells **(A)** and induced cell **(B)**. Regions selected are shown in the optical images in inset in **(B, D)**, respectively. Variation in the intensity of the 1,580 cm^−1^ peak across the cell wall of uninduced **(B)** and induced **(D)** cells along the white line marked in **(A, C)**. The intensity of peak at 1,580 cm^−1^ suggests an increase in concentration of cell wall bound phenolics with induction of switchgrass ST1 suspension cultures using epibrassinolide. High-resolution Raman mapping of the uninduced cell **(E)** reveals variations in the chemical content as shown in the average spectra of selected areas **(F)**, with features (region A) exhibiting traits of ferolic acid (~1,175 and 1,660 cm^−1^).

Optical images, shown as an inset in **Figures 1B, D** for uninduced and induced ST1 suspension cells, respectively, revealed uniform and rounded cells. Variations in phenolics were observed across the cell wall, as indicated by the two-dimensional spectroscopic images of adjacent switchgrass cells which were constructed by integrating the intensity around the phenolic band at 1,580 cm^−1^ (**Figures 1A, C, E**). A 3.5 times increase in intensity of the band was observed in the induced cells compared to the uninduced cells, indicative of lignification, which was also confirmed by the intensity profiles in **Figures 1B, C**. Spectra acquired in regions approaching the cell wall of the induced cells revealed bands at 1,580, 1,600, and 1,630 cm^−1^, suggesting an increase in concentration of lignin derivatives and phenolic acids when compared to the uninduced sample (**Figure 1F**) (Wang et al., 2012). Further quantification and analysis of lignin and lignin-precursors were undertaken using standard methods described hereafter.

### Analysis of Lignin-Precursors in Switchgrass Cell Suspensions Using Standard Methods

Extraction of the cell suspensions using standard methodology resulted in a dry weight sample loss of 15%–18%, due to the removal of non-structural phenolics, cell components, and organelles (Morrison and Stewart, 1995; Fukushima and Hatfield, 2004). The quantification of lignin precursors is reported as extractives-free cell wall bound phenolics (CBP) to account for the various phenolic forms found in the cells (covalent-, ester-, and ether-linked phenolics, etc.). The % CBP as determined by acid hydrolysis for the uninduced and induced switchgrass cells was 18.5 ± 0.7% and 29.9 ± 0.3%, respectively (**Table 1**). The total amount of CBP increased by 62% after inducing lignification with epibrassinolide, which is in agreement with our Raman findings. The content of acid soluble lignin-precursors (ASLP) and acid insoluble lignin-precursors (AILP) was determined using an acid hydrolysis-wet chemistry method. With lignin induction, the AILP content increased from 5.0 ± 0.7% to 19.0 ± 0.9%, which corresponds to a ~3.8 times increase. This suggests a transformation of monolignols to larger lignin polymer with an increase in ß-O-4 linkages and cross-linkages that present fewer bonds with other cell wall constituents, thus forming a lignin-like polymer that is easier to remove during the hydrolysis process. Concurrently, ASLP decreased by 19%, suggesting the formation of oligomer-based lignin polymer in the cells. The content of ASLP in the cells varied from 10.9 in induced cells to 13.5% in uninduced cells, which is comparable to the 9.8% acid-soluble extractives-free lignin content previously reported in 7-day-old hybrid aspen cultures (Christiernin et al., 2005).

**Table 1:** Cell wall characterization of ST1 switchgrass suspension cultures using standard acid-catalyzed hydrolysis and acetyl bromide methodologies.

	Uninduced		Induced		Senesced Biomass^4^	
	(Extractives-free, Dry basis)					
*Acid hydrolysis methodologies^1,3^*						
Structural ash	2.0^b^	(0.1)	0.9^c^	(0.1)	1.7^a^	(0.5)
Total Carbohydrates	78.9^a^	(1.1)	65.2^b^	(0.8)	70.0^b^	(3.1)
Glucose	34.2^b^	(1.4)	20.7^c^	(1.2)	38.4	(1.4)
Xylose	16.7^b^	(1.3)	17.5^b^	(0.9)	24.0^a^	(1.6)
Arabinose	10.3^b^	(0.4)	11.7^a^	(0.3)	3.2^c^	(0.1)
Galactose	9.2^b^	(0.2)	13.8^a^	(0.3)	4.2^c^	(0.4)
Mannose	8.5^a^	(0.1)	1.5^b^	(0.1)	0.2^c^	(0.1)
**Total lignin (CBP)**	**18.5^c^**	**(0.7)**	**29.9^a^**	**(0.3)**	**23.5^b^**	**(1.1)**
ASLP	13.5^a^	(0.3)	10.9^b^	(0.2)	3.2^c^	(0.3)
AILP	5.0^c^	(0.7)	19.0^b^	(0.9)	20.7^a^	(0.8)
*Acetyl bromide methodology,^2,3^*						
**Total lignin (CBP)**	**16.4^b^**	**(1.2)**	**24.2^a^**	**(2.1)**	**23.8^a^**	**(2.0)**

ASLP, acid soluble lignin-precursors; AILP, acid insoluble lignin-precursors. Values are reported in %.

^1^Following NREL protocols for sulfuric acid-catalyzed hydrolysis of biomass with gravimetric analysis of acid insoluble lignin and structural ash after exhaustive extraction, performed in triplicate.

^2^Acetyl bromide CBP determined using methods by (Fukushima and Kerley, 2011) with extinction coefficient of 23.077 g/L.

^3^Letter representation for mean separation by Fisher’s LSD (p = 0.05) comparing compositional values for uninduced cells, induced cells, and senesced switchgrass tissue.

^4^Average of five biological replicates from the field.

In addition to the changes in lignin precursors, the suspension cultures appeared to uptake and retain minor concentrations of inorganics from the media until utilized in the early stages of lignification as structural ash decreased by approximately 55% with induction. Total carbohydrates also decreased from 78.9 ± 1.1% to 65.2 ± 0.8% after induction. Specifically, large decreases (39%) in glucose (from 34.2 ± 1.4% to 20.7 ± 1.2%) and mannose (82.4% from 8.5 ± 0.1% to 1.5 ± 0.1%) content were observed. Minor but statistically significant increases were observed for arabinose and galactose with induction. To allow for comparisons with more mature switchgrass tissue primarily composed of secondary cell walls, a field-grown senesced switchgrass control sample from the same phenotype as the cell cultures was simultaneously analyzed. ASLP in the induced cells was about 3 times higher than in the control, suggesting a very different lignin structure. The carbohydrates monomer analysis revealed that the content of arabinose and galactose was significantly higher in suspension cultures, suggesting the presence of pectin primarily with the middle lamella region and primary cell wall. The concentration of xylose in the induced cell suspension culture was 13% lower than the switchgrass control, implying that xylan synthesis occurs during formation of the secondary cell wall (Christiernin et al., 2005). The glucose content was found to be 42% higher in senesced tissues than in induced cells, indicating the presence of more hemicellulose in the cells than in the mature reference biomass. Overall, the composition analysis indicates that the cells were mostly composed of primary cell walls, with possible partial secondary cell wall formation after 7 days post induction.

Total CBP measured using an acetyl bromide assay (**Table 1**) also confirmed these findings with 16.4 ± 1.2% CBP in uninduced cells compared to 24.2 ± 2.1% for induced cells.

### Analysis of CBP in Switchgrass Cell Suspensions Using Py-GC/MS

During the analytical pyrolysis of lignocellulosic material many factors contribute to the composition of eluting gas vapors as a result of the complex pyrolysis reaction pathways. For example, depolymerization of glycosidic units in cellulose forms levoglucosan as the predominant pathway for cellulose. However, the production of levoglucosan is inhibited by the presence of alkali metals (sodium and potassium) with disruption of the cross-linking between chains (trans-glycosylation), resulting in furfural derivatives and other lower molecular weight carbonyl compounds. Additionally, the pyrolysis pathways of cellulose have an impact on pyrolysis of other biomass constituents such as lignin and xylan (Evans and Milne, 1987; Sykes et al., 2009). Therefore, various gas fragments eluting during Py-GC/MS analysis were expected to vary between the pre- and post-induction suspensions, primarily due to the variations in chemical composition, in particular structural ash (**Table 1**). However, a careful review of pyrograms for determination of the origin of the peaks as lignin, cellulose, or hemicellulose derivatives provides a chemical fingerprint that can be used quantitatively to estimate changes in concentration or structure of cell wall components from the peak intensities (Alves et al., 2006; Fahmi et al., 2007; Sykes et al., 2009). A summary of common peak assignments used in analytical pyrolysis of biomass is provided in **Table 2**.

**Table 2:** Mass spectrum peak assignments associated with pyrolysis mass spectrometry for switchgrass samples.

m/z	Assignment	S,G,H precursor
57, 73, 85, 96, 114	C5 sugars	–
57, 60, 73, 98, 126, 144	C6 sugars	–
94	Phenol	H, S, G
109	Guaiacol	G
120	Vinylphenol (*p*-coumaric acid)	H, S, G
123	4-Methyl guaiacol	G
124	Guaiacol	G
137	Coniferyl alcohol/propyl guaiacol	G
138	4-methyl guaiacol	G
139	Syringol	S
147	*p*-coumaric acid	–
149	(Iso)Eugenol	G
150	Vinylguaiacol/ferulic Acid	G
151	Vanillin	G
152	4-ethylguaiacol, vanillin	G
153	4-methylsyringol	S
154	Syringol	S
164	*p*-coumaric Acid, (iso)eugenol	–
165	Sinapylaldehyde, vinylsyringol	S
167	Sinapyl alcohol, acetyl guaiacol	S, G
168	4-methylsyringol	S
178	Coniferaldehyde	G
180y	4-vinylsyringol/coniferyl alcohol	S, G
182	Syringaldehyde	S
194	4-propenylsyringol/ferulic acid	S
208	Sinapyladehyde	S
210	Sinapylalcohol	S
135, 133, 128, 123, 121, 117, 113, 110, 109, 102, 100, 98, 95, 91, 86, 84, 82, 81, 80, 74, 68	Other nonstructural components	–

m/z, mass:charge ratio of fragments extracted; major lignin peaks assignments: syringyl (S), guaiacyl (G), and p-hydroxyphenyl (H).

**Figure 2:**
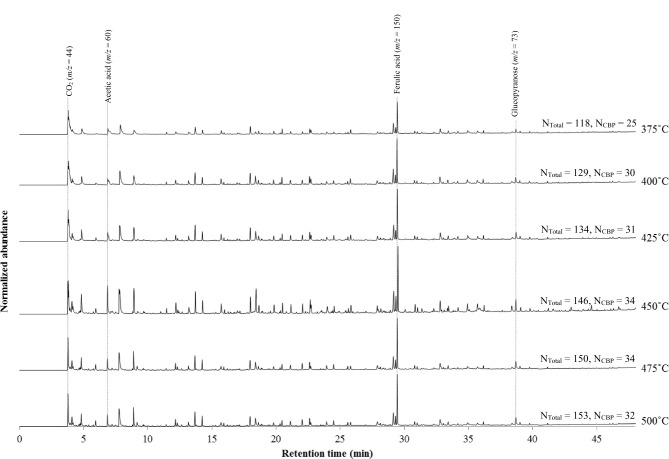
Py-GC/MS pyrograms of 7-day induced ST1 switchgrass suspension cells with various pyrolysis temperatures; N_Total_, number of structural peaks; and N_CBP_, number of peaks with *m/z* values associated with chemically bound phenolics (**Table 3**).

To select the optimum pyrolysis conditions for the dried and extractives-free suspension cells, pyrolysis temperatures ranging from 375°C to 500°C in 25°C increments were tested, looking for elution of an increased number of gas fragments associated with cell wall components with few thermal degradation peaks consisting of small gas fragments. Higher temperatures were not tested in order to avoid additional cracking of the primary pyrolysis vapors (Evans and Milne, 1987). As seen in **Figure 2**, minimal peak resolution and 118 detected structural peaks indicate an incomplete pyrolysis with an experimental temperature of 375°C. The intensity of characteristic peaks of p-vinylguaiacol/ferulic acid (*m/z* = 150), acetic acid (*m/z* = 60), and the fragment ion of carbohydrates (*m/z* = 73) increased when approaching 450°C. However, these peaks decreased in intensity while small molecular weight gas fragments increased in abundance as pyrolysis temperatures rise to 475°C and 500°C. In particular, an increase of the CO_2_ peak (*m/z* = 44) is indicative of the degradation of both carbohydrates and phenolic constituents in the cells. Therefore, a temperature of 450°C for 12 s prior to injection to the GC/MS was determined to be the optimum condition for the pyrolysis of our cells.

**Figure 3:**
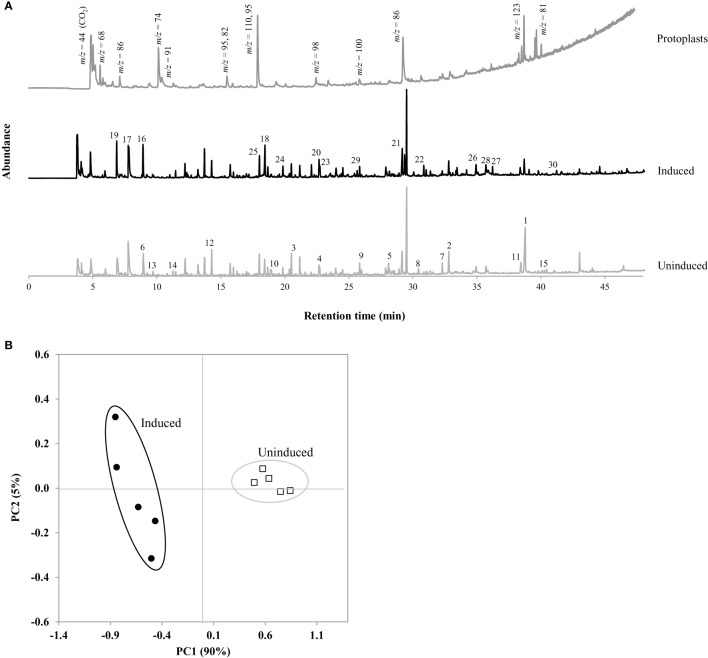
**(A)** Py-GC/MS pyrograms of ST1 switchgrass protoplasts, and extracted induced and uninduced suspension cells. Protoplast gas fragment peaks identified by mass:charge ratio (*m/z*). **(B)** PCA scores plot obtained by principal component analysis (PCA) of integrated peak areas with tentative identifications listed with loadings values in **Table 5**. Numbered peaks were determined as more abundant in each suspension culture sample.

Prior to analyzing the Py-GC/MS datasets, removal of nonstructural peaks was achieved by comparing suspension cell pyrograms to that of switchgrass protoplasts produced to act as control for identification of gas fragments that did not originate from cell wall constituents. As seen in **Figure 3A**, pyrograms for ST1 switchgrass protoplasts exhibited peaks with unknown structure or origin. These *m/z* values were thus assumed to be related to media or intracellular components. Additionally, an exhaustive extraction process with a mixture of aqueous and organic solvents provided a homogenous, clean sample with little background interference by removing non-structural phenolics that may be erroneously interpreted as part of the developing lignin molecule, and allowing for simplified quantification and characterization of lignin precursors using Py-GC/MS. Standard peak assignments based on *m/z* for cell wall components are listed in **Table 2** with suggested origin of phenolic compounds as derived from either S-, G-, or H-lignin.

A compilation of specific phenolic peaks shared in all samples or found uniquely in either uninduced or induced cells is presented in **Table 3**. While the majority of these shared peaks are G-derivative CBP, all the cells contain basic phenol units (retention time (RT) = 22.067 min, *m/z* 94), vinylphenol (RT = 29.340 min, *m/z* 120), 4-allyl phenol (RT = 32.027 min, *m/z* 134), and 5-methylfurfural (RT = 33.357 min, *m/z* 110). Possible derived hydroxycinnamic acid peaks included 4-vinyl guaiacol/ferulic acid (RT = 29.510 min, *m/z* 150), *p*-coumaric acid (RT = 33.848 min, *m/z* 147) and eugenol (RT = 37.058 min, *m/z* 164). While few S-derived lignin precursors were observed in the uninduced cells, many CBP peaks associated with S-type precursors were found in the induced cells including: syringol (RT = 30.851 min, *m/z* 154), 4-methyl syringol (RT = 33.107 min, *m/z* 168), 4-vinyl syringol (RT = 36.224 min, *m/z* 180), 4-propenylsyringol (RT = 36.639 and 39.075 min, *m/z* 194), aceto syringone (RT = 41.231 min, *m/z* 196), and sinapylaldehyde (RT = 42.886 min, *m/z* 208).

**Table 3:** Phenolic peaks found in pyrograms of uninduced and induced switchgrass suspension cells.

*Phenolic peaks found in both induced and uninduced switchgrass suspension cells*			
**RT (min)**	***m/z***	**Tentative identification**	**Origin**
22.067	94	Phenol	S/G/H
23.503	108	2-methyl phenol	G
24.518	108	2-methyl phenol	G
25.639	138	4-methylguaiacol	G
26.894	152, 137	Vanillin, coniferyl alcohol	G
27.960	152, 137	4-ethyl-guaiacol	G
28.565	152, 137	Vanillin, coniferyl alcohol	G
29.340	120	4-vinylphenol	PCA
29.510	150	4-vinyl guaiacol/ferulic acid	G/FA
32.027	134	4-allyl phenol	H
33.357	110	5-methyl furfural	S/G/H
33.447	151	Vanillin	G
33.772	152	4-ethyl guaiacol/vanillin	G
33.848	147	*p*-coumaric acid	PCA
33.988	206	Di-butyl phenol	S
37.058	164	Eugenol	G
***Phenolic peaks uniquely found in uninduced switchgrass suspension cells***			
**RT (min)**	***m/z***	**Tentative identification**	**Origin**
11.438	165, 108	Vinyl syringol, m-cresyl methylcarbamate	S, G
14.054	104	phenylethyl methacrylate	H
22.152	120	tert-butyl phenyl carbonate	H
25.124	138	4-methyl guaiacol	G
31.316	164	4-ethenyl-1,2-dimethoxy-benzene	G
34.262	147	*p*-coumaric acid	PCA
34.362	124	Guaiacol	G
42.125	147	*p*-coumaric acid	PCA
***Phenolic peaks uniquely found in induced switchgrass suspension cells***			
**RT (min)**	***m/z***	**Tentative identification**	**Origin**
22.668	124	Guaiacol	G
30.851	154	Syringol	S
31.762	138	4-methyl guaiacol	G
33.107	168	4-methyl syringol	S
35.258	138, 137	Propyl guaiacol/4-methyl guaiacol	G
35.383	167	Acetyl guaiacol	G
36.224	180	4-vinyl syringol	S
36.534	137	2-propyl guaiacol	G
36.639	194	4-propenyl syringol	S
39.075	194	4-propenyl syringol	S
39.485	208	Sinapylaldehyde	S
40.940	150	Ferulic acid	FA
41.231	196	Acetosyringone	S
41.566	180	Coniferyl alcohol	G
42.886	208	Sinapylaldehyde	S
43.947	167	Syringal acetone	S

RT, retention time; m/z, mass:charge ratio of fragments extracted; major lignin peak Origins: syringyl (S), guaiacyl (G), p-hydroxyphenyl (H), ferulic acid (FA), and p-coumaric acid (PCA).

Py-GC/MS provides a chemical fingerprint that is unique to the composition of the samples as the presence or absence of individual peaks can aid in characterization of the chemical composition. Representative pyrograms for uninduced and induced ST1 switchgrass cells with these significant peaks are shown in **Figure 3A**. The analysis of the suspension cultures required ~200 peaks for comprehensive analysis of the cell wall, determined as significant by meeting a designated signal to noise ratio (5:1). Next, multivariate statistical analysis was employed to detect variations within these large and complex data sets. PCA is a descriptive method that allows for visualization of variability within a large data set, thus identifying trends, differences, or similarities within samples. PCA transforms the multivariate data set into a new data set that is dependent on new variables, called principal components (PCs). The first PC contains the most variation in the pyrogram and is associated with a set of loadings, which are directly related to contributing evolved peaks in the pyrogram. Each pyrogram has an associated score on each PC. Plotting the scores of different PCs against one another reveals pyrograms with similar score indicating that they possess similar composition (**Figure 3B**). In this case, the cells separate by induction along PC1 (90%). As seen in **Table 4**, significant gas fragments contributing to the sample clusters were classified using the *m/z* in conjunction with a NIST library to distinguish between fragments originating from structural carbohydrates, lignin, or non-structural compounds derived from either the samples or growth media (Sykes et al., 2008; Mann et al., 2009; Sykes et al., 2009; Penning et al., 2014). Given the larger spread within the induced cluster, it can be assumed that there is more variability in the composition of the induced samples, suggesting a degree of natural variability in the efficacy of the induction after 7 days. Peaks possessing a positive score for PC1 were found more prominently in the uninduced cells and are listed in **Table 4** (peaks 1–15) in order of significance. The peak with the largest difference (85.7%) before and after induction represents fragment ions of carbohydrates (38.764 min, *m/z* 73). The abundance of this compound and other C6 sugars peaks in the loadings supports the significantly higher glucose content in the cells found during wet chemistry analysis (**Table 2**). The remaining peaks were also derivatives of carbohydrates in the cells, implying a higher initial cellulose and hemicellulose content in the primary cell wall of the uninduced cells compared to the developing induced cells with lignin initiation, again supported by the previously determined total carbohydrates content in the cells (**Table 2**). In the same manner, peaks associated with a negative score for PC1 reveal those gas fragments that are more abundant in the induced cells (**Table 4**, peaks 16–30). Acetic acid was found more abundantly in the induced cells (RT = 7.777 and 6.901 min, *m/z* 60), suggesting the presence of more C5 sugars after lignin induction and concurrent formation of the secondary cell wall (Christiernin et al., 2005). The distribution of carbohydrate peaks in the loadings for both uninduced and induced cells shows products from both the glycosidic rupture mechanism (depolymerization, resulting in *m/*z 126, 43) and transglycosylation rupture mechanism (alkali-metal catalyzed pathway, characterized by the presence of *m/*z 144, 60) of cellulose (Evans and Milne, 1987).

**Table 4:** Principal component analysis (PCA) loadings table comparing gas fragments resulting from pyrolysis of extracted uninduced and induced ST1 switchgrass suspension cells.

Peak No.	RT (min)	m/z	ID	CW Origin	Origin
					(S,G,H)
*Peaks more abundant in uninduced cells*					
1	38.764	73, 60	1,6-anhydro-.beta.-D-glucopyranose	C6	–
2	29.510	150	Ferulic acid	–	FA
3	32.807	73, 57	Sucrose	C	–
4	20.517	114, 58	Unknown	C5	–
5	8.942	74, 43	Methyl ester acetic acid	C5	–
6	29.150	73,57	Unknown	C	–
7	22.663	128, 124, 43	Guaiacol	C	–
8	28.095	85, 69, 43	Unknown	C5	–
9	32.291	144, 97, 87	Unknown	C6	–
10	14.279	96, 39	Furfural	C5	–
11	38.434	73,43	1,6-anhydro-alpha-D-galactofuranose	C5	–
12	30.441	126, 97, 69	5-(hydroxymethyl)-2-furancarboxaldehyde	C5	–
13	21.162	112, 55	2-hydroxy-3-methyl-2-cyclopenten-1-one	C5	–
14	25.994	142	3,5-dihydroxy-2-methyl-4H-pyran-4-one	C	–
15	18.941	114, 86, 43	Unknown	C5	–
*Peaks more abundant in induced cells*					
16	8.917	43	Acetic acid methyl ester	C5	–
17	18.421	70, 42	3-methyl-hexanal	B	–
18	22.668	124, 109, 81	Guaiacol	L	G
19	29.340	120	4-vinylphenol (p -coumaric acid)	–	pCA
20	7.777	60, 43	Acetic acid	C5	–
21	30.851	154, 139, 85	2,6-dimethoxy-phenol, Syringol	L	S
22	6.901	60, 43	Acetic acid	C5	–
23	22.067	94, 66	Phenol	L	S/G/H
24	36.224	180, 165, 137	4-vinylsyringol	L	S
25	19.841	84, 55	2-methyl-2-butenal	B	–
26	41.231	196, 181	Acetosyringone	L	S
27	39.075	194, 91	4-propenyl syringol	L	S
28	34.938	116, 73, 45	1-(acetyloxy)-2-propanone	C	–
29	33.452	152, 151	Vanillin	L	G
30	41.556	180	Coniferyl alcohol	L	G

Gas fragment peaks identified by retention time (RT), mass:charge ratio (m/z), tentative assignment and origin within the cell wall (C5, derivative of five carbon carbohydrate in hemicellulose; C6, six carbon carbohydrate; C, unidentifiable carbohydrate; B, unidentifiable component from either carbohydrates or lignin) and origin from chemically bound phenolic (CBP) precursor (G, guaiacyl lignin; S, syringyl lignin; H, p-hydroxyphenyl lignin; FA, ferulic acid; pCA, p-coumaric acid).

As expected, the majority of subsequent loadings peaks found more abundant in the induced cells is related to the development of CBP derived from lignin, including: guaiacol (RT = 22.668 min, *m/z* 124), syringol (RT = 30.851 min, *m/z* 154), 4-vinyl syringol (RT = 36.224 min, *m/z* 180), and aceto syringone (RT = 41.231 min, *m/z* 196). Formation of the secondary cell wall was supported with the increased presence of these S-lignin precursors bound to the cell wall, which also demonstrates a shift in the S/G ratio within the cell with more G- and S- lignin precursors expected in the cells after induction.

As seen in **Table 5**, total CBP content for the extractives-free, uninduced switchgrass suspension cells was estimated at 13.6 ± 0.2%, adjusted to 13.3 ± 0.2% when accounting for the minor ash content in the cells. The impact of additional cell wall components such as ash should be taken into consideration as the catalytic properties of the inorganics found in the cell wall and cell growth media may have a considerable influence on the pyrolytic pathways of cellulose and lignin, in particular if ash is found in significant concentration in the sample (Evans and Milne, 1987). However, in these samples, ash was a minor component (≤2.0%, **Table 1**). The pyrolysis gas fragments were assigned as originating from 1.4 ± 0.6%, 15.7 ± 1.9%, and 6.6 ± 0.8% for S-, G-, and H-type lignin precursors, respectively. The calculation of % H-type lignin precursors also included phenolic peaks unidentified in origin (S/G/H). The majority of peaks were assigned to ferulic acid (71.0 ± 1.6%) and *p*-coumaric acid (5.3 ± 1.3%). Total % CBP in the induced cells was calculated as 32.6 ± 1.3% when corrected for ash and carbon dioxide produced during pyrolysis. The percentage of syringyl precursor units in induced cells rose to 21.3 ± 1.2%, while G-type precursors rose to 27.0 ± 0.5%, supporting the shift in S/G ratio previously described by PCA. The amount of ferulic and *p*-coumaric acids decreased to 34.6 ± 1.1% and 1.4 ± 0.2%, respectively. Theoretically, after induction with epibrassinolide, these bound phenolic acids act as nucleation sites for the formation of more complex lignin polymers with ether-linked hydroxycinnamic acids, especially ferulic acid, forming bridges between lignin and polysaccharides by also being ester-linked to sugars, coupling with monolignol radicals forming inter-unit linkage types, essentially allowing ferulate to act as monolignol (Lam et al., 2001). Alternatively, ferulic and *p*-coumaric acids could be utilized within the biosynthetic pathway to produce additional S-type lignin monomers within the formation of the secondary cell wall. More specifically, the increasing number of syringyl-derived gas fragments may be explained by the order of monomer polymerization in the cells (Terashima et al., 1996). Initial monolignol chains supplied to the lignifying cell wall are theorized in order of H, G, and S, with phenolics located in the cell corners and compound middle lamella region composed of primarily H and G-lignin units, while the secondary cell wall has increasing G and S content. The composition of the CBP for both cell types was confirmed by nitrobenzene oxidation, demonstrating how Py-GC/MS could facilitate studies of lignin biosynthesis without the need for separate analytical techniques such as chromatography.

**Table 5:** Cell wall characterization by Py-GC/MS for percentage of chemically bound phenolics (CBP) derived from either syringyl (S), guaiacyl (G), *p-*hydroxyphenyl (H) monolignols, and ferulic (FA) and *p*-coumaric (*p*CA) acids, Py-GC/MS correction factor (CF), and nitrobenzene oxidation.

	% CBP (extractives-free, dry basis)^1^	Py-GC/MS						Nitrobenzene Oxidation				
		CF^2^	%S	%G	%H^3^	%FA	%pCA	%S	%G	%H	%FA	%pCA
Uninduced	13.6 (0.2)	1.36										
(corrected for ash)	13.3 (0.2)	1.39	1.4 (0.6)	15.7 (1.9)	6.6 (0.8)	71.0 (1.6)	5.3 (1.3)	5.3 (1.0)	22.4 (3.8)	2.1 (2.5)	65.7 (5.7)	2.5 (1.9)
**(CO_2_ Inclusive)**	**19.6 (0.9)**	**0.94**										
Induced	21.2 (1.2)	1.41										
(corrected for ash)	21.0 (1.2)	1.42	21.3 (1.2)	27.0 (0.5)	15.8 (0.6)	34.6 (1.1)	1.4 (0.2)	24.1 (3.1)	33.6 (3.2)	12.8 (2.1)	27.0 (3.4)	3.5 (0.9)
**(CO_2_ Inclusive)**	**31.6 (1.3)**	**0.95**										

Standard deviation is shown in parentheses.

^1^%CBP calculated as the percentage of lignin precursor peaks (see **Tables 2** and **3**) within the total of structural peaks, with corrections for ash in the cells and inclusion of the carbon dioxide peak.

^2^CF, correction factor calculated for the subsequent Py-GC/MS analyses to account for non-accounted phenolics lost as CO_2_ during the pyrolysis process as the ratio of phenolics determined by acid hydrolysis methods to that determined with Py-GC/MS.

^3^%H, chemically bound phenolics both derived from H-type lignin precursors as well as those unidentified in origin (S/G/H).

When comparing the quantification of % CBP by Py-GC/MS with the two standard validation methods, acetyl bromide and conventional wet chemistry, Py-GC/MS analysis resulted in the highest estimation of lignin precursors for both cell types, most likely due to slight overestimations for these peaks when correcting for carbon dioxide as both carbohydrates and phenolics degradation products are represented in this peak. Therefore, the Py-GC/MS correction factors were determined as 0.94 (uninduced cells) and 0.92 (induced cells) when comparing the ratio of % CBP from Py-GC/MS to the one found using the wet chemistry acid hydrolysis method. Therefore, subsequent analysis of phenolics within similar switchgrass cells should be reduced by 6%, assuming the standard methods are accurately measuring lignin content in the cells. Without inclusion of the CO_2_ peak, the % CBP calculated from Py-GC/MS data was underestimated by approximately 39%–42%. By utilizing either the CO_2_ peak in CBP determination or applying the 1.39 (uninduced) or 1.42 (induced) correction factor, the % CBP can be accurately measured using this technique when compared to acid hydrolysis standard methodology. For example, % CBP in the uninduced cells was calculated as 19.6 ± 0.9% by Py-GC/MS (**Table 5**) and 18.5 ± 0.7% by acid hydrolysis (**Table 1**), or a difference of 5.6%. Similarly, % CBP in the induced cells was calculated as 32.6 ± 1.3% (Py-GC/MS) and 29.9 ± 0.3% (acid hydrolysis), a difference of 8.3%. Using the acetyl bromide method, the uninduced cells were found to contain 16.4 ± 1.2% CBP, and 24.2 ± 2.1% in the induced cells. Relative comparisons of these three techniques for quantification of CBP indicate the cells possess 66.3% (Py-GC/MS), 61.6% (acid hydrolysis), and 47.6% (acetyl bromide) more phenolics after induction. While normally an acceptable quantification method for lignin in whole biomass, the acetyl bromide determination of % CBP was significantly lower for both cell types, demonstrating this method’s weakness for accurate quantification of phenolics in these cells. Interestingly, Eberhardt et al. (1993) have reported that the acetyl bromide method overestimates lignin/phenolics content in cell suspension cultures of *Pinus tuedu*. Therefore, acetyl bromide determination should be recommended for relative screening applications only. If absolute quantification of lignin/phenolics content is needed, we recommend obtaining a correction factor using the wet chemistry acid hydrolysis method. However, if Py-GC/MS is to be employed for relative assessment of lignin/phenolic compounds in different treatments/samples, a correction factor may not be necessary for conducting the analysis and comparison. We note that compared to other previously published Py-GC/MS studies (Lopes et al., 2011; Gerber et al., 2016), we utilized extractives-free cell wall samples for our Py-GC/MS analysis. It has been shown that extractives, constituting the non-structural components within the cytoplasm of the cells, can interfere with quantification of lignin and other cell wall components (Eberhardt et al., 1993; Thammasouk et al., 1997; Hames, 2009; Burkhardt et al., 2013). Hence, we removed the extractives from the samples before conducting compositional analysis of lignin by Py-GC/MS.

Of interest is the high hydroxycinnamic acid content in the switchgrass cells prior to induction for lignification, with approximately 76% of the CBP composition consisting of fragments related to ferulic and *p*-coumaric acids. The post-induction fraction of hydroxycinnamic acid was significantly decreased to ~35% of the total CBP. One possible explanation includes the utilization and conversion of these hydroxycinnamic acids to various precursors along the pathways to monomers during biosynthesis of the lignin molecule. Previous work has discussed that lignins are naturally partially acylated due to incorporation of lignin monomers enzymatically preacylated by various acids, including *p*-coumarates, in C4 grasses (Withers et al., 2012). *P*-coumarates are not incorporated into polymerized lignin chains, remaining almost entirely as free-phenolic pendent entities bound primarily to lignin, with smaller portions bound to carbohydrates (Dobberstein and Bunzel, 2010; Wilkerson et al., 2014). However, monolignol *p*-coumarate conjugates are compatible with traditional monolignols polymerization reactions and therefore act as a lignin “monomer” for lignification in grasses (Wilkerson et al., 2014). The alternative pathways for formation of these monolignol *p*-coumarate conjugates are discussed elsewhere (Withers et al., 2012). Ferulate (with an additional methoxyl group) can be integrated into the lignin polymer, leading to carbohydrate-lignin bridges, with an initial ester-linkage to hemicelluloses (arabinoxylans) or pectins and then ether linked to lignin, allowing for the formation of phenolic polymers by providing attachment sites to cell wall polysaccharides for later lignification (De Ascensao and Dubery, 2003). The interaction of ferulates with lignin monomers and oligomers is complex, but it is believed that ferulate is ether-linked to either the β- or benzyl position of lignin. Ferulate radicals can also couple with monolignol radicals to form inter-unit linkage types such as β-5 (phenylcoumaran type), β-β (pinoresinol type), 5-5 (binphenyl type) (Wilkerson et al., 2014). These are hypothetical theories on the synthesis of lignin and outside the scope of this study. However, work by many in the field has been conducted to understand these mechanisms (Scalbert et al., 1985; Kondo et al., 1990; Campbell and Ellis, 1992; Lam et al., 2001; Singh et al., 2005; Withers et al., 2012; Wilkerson et al., 2014) and the Py-GC/MS method described here could be used to further examine changes in hydroxycinnamates and lignin synthesis in cell culture cells and plant tissues developing primary and secondary walls.

## Conclusions

### Establishment of Py-GC/MS for Analysis of Suspension Cells

A major conclusion from this work is the validation of the Py-GC/MS as a viable technique to quantify the composition of suspension cells. This is confirmed by the comparison of CBP content determined by widely accepted methods including acetyl bromide solubilization and wet chemistry with an acid-catalyzed hydrolysis. Py-GC/MS phenolic values calculated as the sum of lignin pyrolysis products divided by the sum of all structural fragments originating from carbohydrates and lignin (after removal of non-structural component fragments) is not a measure of the absolute value of lignin. However, a correction factor was calculated for switchgrass through comparison of Py-GC/MS lignin and total lignin content as determined by the wet chemical technique (sum of acid-insoluble and acid-soluble lignin on an extractives-free basis) (Sykes et al., 2009). Future analyses can therefore be completed in confidence that the measurements made are accurate and comparable to previously utilized techniques for suspension cultures or small aggregate cell samples.

We note that Py-GC/MS run times average ~55 min per sample in this work and lack automation of sample preparation and data analysis to date. In addition, the complexity of Py-GC/MS datasets calls for the implementation of new data analyses packages to increase the high-throughput capability of this technique (Smith et al., 2006). However, Py-GC/MS offers the advantage of being an “all-in-one” approach for providing quantitative and qualitative information of plant cell walls without extensive sample preparation or processing with hazardous chemicals. Hence, Py-GC/MS could be used for conducting quick and reliable cell wall compositional studies, including the quantification of S, G, and H units without the isolation of the lignin fraction, selection of an appropriate standard, or additional external instrumentation such as chromatography. In time, Py-GC/MS analysis could also become advantageous for the identification of specific peaks thanks to the additional time dimension with chromatographic separation of evolved gas fragments, thus providing more information surrounding the general lignin composition in early developmental tissues. There are other methods such as thioacidolysis (Harman-Ware et al., 2016), pyrolysis-molecular beam mass spectrometry (Sykes et al., 2009; Penning et al., 2014) and nuclear magnetic resonance technology (Hedenström et al., 2009; Kim and Ralph, 2010; Karlen et al., 2017) that use relatively less tissues, about 2 mg, 4–20 mg and 20–100 mg of tissues, respectively, for cell wall compositional analyses. The requirement for sample mass (250 µg) far inferior to the scale of milligrams to grams of dried cells required for conventional methods and the relative ease of conducting the analysis using Py-GC/MS constitutes a unique opportunity to enable comprehensive cell wall compositional analysis with less tissue samples. The method presented here could be adapted for translational genomics or for fundamental studies focused on understanding the molecular and physiological mechanisms involved in regulating biomass recalcitrance.

### Biological Significance

This method was used in the exploration into the growth of cell cultures, in particular with the development of the secondary cell wall and synthesis of lignin. The technique facilitates characterization of lignin. In this case, we determined that the uninduced cell culture possessed lignin precursors, consisting mostly of ferulic acid and guaiacyl units. After induction, the cells were found to contain syringyl units, supporting the development of the secondary cell wall. These results resemble other studies with similar growth conditions in that most likely, fewer β-O-4’ bonds were present in early plant suspension cultures and the lignin that is deposited contains an abundance of guaiacyl units until later growth stage results in the synthesis of syringyl units (Christiernin et al., 2005). However, Py-GC/MS is limited in its ability to address whether the detected phenolics are more of a polymeric lignin in the traditional sense as opposed to other forms of wall-bound phenolics. We show that when used in conjunction with multivariate statistical analyses (PCA), Py-GC/MS datasets can distinguish cell wall residues within the same species, allowing for screening acute differences in cell wall chemistry, particularly in transgenic material, to investigate results of cell wall gene transformations. Statistical analysis of this information allows for rapid visual comparisons of the pyrolysis products, including bound hydroxycinnamic acids. When screening cell wall genes for applications in the development of novel feedstocks for biofuels and value-added coproducts, it is important to note that in grasses, cell wall-bound *p*-coumaric and ferulic acids increased the recalcitrance of the biomass, similar to the effect of lignin affecting digestion of cell wall polysaccharides (Kondo et al., 1990; De Ascensao and Dubery, 2003). The *p*-coumaric and ferulic acids could be incorporated into hemicelluloses leading to cell wall rigidification and likely contributing to biomass recalcitrance (Hatfield et al., 2017; Lapierre et al., 2018). Further research will be necessary to evaluate which components of the cell walls incorporate the *p*-coumaric and ferulic acids and their relative impact on cell wall rigidification and biomass recalcitrance.

This work has shown that accurate measurements can be obtained using Py-GC/MS on biomass cells for characterization of lignin-precursors through analysis of a unique chemical fingerprint for each sample. In this case, we used a suspension culture that appeared to have some spontaneity in deposition of lignin-like molecules on the cell wall; however, with an elicitor to induce lignification, we used this switchgrass culture as a model system for lignin biosynthesis in grasses. We were able to test the potential of Py-GC/MS as a rapid characterization technique for samples of this nature, especially when used in conjunction with multivariate statistics to identify changes in chemical composition through eluting gas vapors during pyrolysis. Therefore, our future work will consider Py-GC/MS as a standardized quantification technique in the construction of multivariate prediction models with high-throughput spectral instruments, such as Fourier transform infrared spectroscopy, to clearly establish its usefulness in the development of new cell cultures for application in the bioenergy and bio-based chemical industries. Future work estimating the appropriate growth conditions and duration required to develop a tissue culture system that reflects developmental processes occurring in the resulting plants would prove invaluable. This would allow for more rapid selection of transgenic lines prior to regeneration of whole plants. In addition, this system will enable the dissection of mechanisms involved in monolignol transport and lignin biosynthesis. For instance, it would be interesting to test whether the CBP changes detected belong to the cytoplasm or to the cell wall matrix, where the necessary oxidizing enzymes are present and active. A mechanistic understanding of CBP production and lignin synthesis could further improve the capabilities to selectively transform potential feedstocks like switchgrass which are utilized in the development of biofuels and chemicals.

## Data Availability Statement

All datasets presented in this study are included in the article.

## Author Contributions

LK carried out characterization of the cells, including all statistical analyses. JB was responsible for the growth and maintenance of cell cultures, MS and LT performed Raman imaging analysis. SL, CS, TR, and NL conceived the study and participated in its design and coordination. LK, SL, and PV interpreted the results and were responsible for writing the manuscript. All authors contributed to the article and approved the submitted version.

## Funding

This research was supported by Advanced Research Projects Agency – Energy (ARPA-E) Award No. DE-AR0000313.

## Conflict of Interest

The authors declare that the research was conducted in the absence of any commercial or financial relationships that could be construed as a potential conflict of interest.

## Abbreviations

CBP, Cell wall bound phenolics; Py-GC/MS, Pyrolysis-Gas Chromatography/Mass Spectrometry; *m/z*, Mass to charge ratio; RT, Retention time; S, Syringyl lignin unit; G, Guaiacyl lignin unit; H, *p-*hydroxyphenyl lignin unit; HPLC, High-performance liquid chromatography; GPC, Gel permeation chromatography; PCA, Principal component analysis; ASLP, Acid-soluble lignin-precursor; AILP, Acid-insoluble lignin-precursor.
